# Hyperoxia can Induce Lung Injury by Upregulating AECII Autophagy and Apoptosis Via the mTOR Pathway

**DOI:** 10.1007/s12033-023-00945-2

**Published:** 2023-11-08

**Authors:** Yingcong Ren, Song Qin, Xinxin Liu, Banghai Feng, Junya Liu, Jing Zhang, Ping Yuan, Kun Yu, Hong Mei, Miao Chen

**Affiliations:** 1 Department of Critical Care Medicine, Affiliated Hospital of Zunyi Medical University, Zunyi, 563000 Guizhou China; 2 Department of Critical Care Medicine, Zunyi Hospital of Traditional Chinese Medicine, Zunyi, 563000 Guizhou China

**Keywords:** Hyperoxic acute lung injury, Type II alveolar epithelial cells, mTOR, Autophagy, Apoptosis

## Abstract

Oxygen therapy is a crucial medical intervention, but it is undeniable that it can lead to lung damage. The mTOR pathway plays a pivotal role in governing cell survival, including autophagy and apoptosis, two phenomena deeply entwined with the evolution of diseases. However, it is unclarified whether the mTOR pathway is involved in hyperoxic acute lung injury (HALI). The current study aims to clarify the molecular mechanism underlying the pathogenesis of HALI by constructing in vitro and in vivo models using H_2_O_2_ and hyperoxia exposure, respectively. To investigate the role of mTOR, the experiment was divided into five groups, including normal group, injury group, mTOR inhibitor group, mTOR activator group, and DMSO control group. Western blotting, Autophagy double labeling, TUNEL staining, and HE staining were applied to evaluate protein expression, autophagy activity, cell apoptosis, and pathological changes in lung tissues. Our data revealed that hyperoxia can induce autophagy and apoptosis in Type II alveolar epithelial cell (AECII) isolated from the treated rats, as well as injuries in the rat lung tissues; also, H_2_O_2_ stimulation increased autophagy and apoptosis in MLE-12 cells. Noticeably, the experiments performed in both in vitro and in vivo models proved that the mTOR inhibitor Rapamycin (Rapa) functioned synergistically with hyperoxia or H_2_O_2_ to promote AECII autophagy, which led to increased apoptosis and exacerbated lung injury. On the contrary, activation of mTOR with MHY1485 suppressed autophagy activity, consequently resulting in reduced apoptosis and lung injury in H_2_O_2_-challenged MLE-12 cells and hyperoxia-exposed rats. In conclusion, hyperoxia caused lung injury via mTOR-mediated AECII autophagy.

## Introduction

Oxygen therapy is one of the most common clinical treatments used to improve respiratory failure and hypoxia metabolism in tissues and organs [1]. However, oxygen therapy is not completely harmless and can lead to hyperoxic acute lung injury (HALI), which may be complicated by acute respiratory distress syndrome and respiratory failure [2]. The pathogenesis of HALI is complex, which includes oxidative stress [3], cellular autophagy and apoptosis [4, 5], and inflammatory response [6, 7]. However, the exact mechanism contributing to the pathogenesis of HALI remains elusive. It is essential to deepen understanding to identify effective clinical prevention for HALI.

Type II alveolar epithelial cells (AECII) are key structures of the distal pulmonary epithelium and function as intrinsic immune and progenitor cells, participating in pulmonary epithelial repair and regeneration [8, 9]. Therefore, AECII function impairment in response to different stimulation is closely related to lung injuries, including HALI [10]. It is now generally accepted that the key factor in HALI is the production of reactive oxygen species (ROS), which plays an important role in oxidative stress and are directly cytotoxic. This ultimately leads to alveolar-capillary barrier dysfunction, epithelial cell death, and accumulation of neutrophils and other inflammatory cells leading to lung injury [11, 12]. One mechanism is that excess ROS can induce apoptosis [13]. In addition, hyperoxia may also promote apoptosis by initiating exogenous and endoplasmic reticulum apoptotic pathways [14]. Hyperoxia-induced excess ROS production can also induce autophagy [15] which is one of the ways to maintain the stability of the intracellular environment through phagocytosis and degradation of intracellular macromolecules by its lysosomes and their recycling [16].

Autophagy and apoptosis are both programmed cell death and are associated with pathogenesis of various diseases [17, 18]. Through intracellular degradation and reuse of aged and damaged organelles, autophagy can relieve cellular stress and inhibit apoptosis [19]. However, autophagy may also promote apoptosis and thus aggravate the damage through “self-phagocytosis” [20]. The reasons for this contrast are not clear but may be related to the change of autophagic flow and the different effects of autophagy-regulating drugs at different time points [21].

The activity of autophagy and apoptosis can be regulated by various signaling pathways, including the PI3K/Akt/mTOR signaling pathway [22]. When mTOR is activated, phosphorylated-mTOR (p-mTOR) is upregulated, which can maintain autophagy at a low level by inhibiting the formation of autophagic complexes [23]. The PI3K/Akt/mTOR signaling pathway can be involved in the regulation of various physio-pathological mechanisms, such as acute lung injury, by altering the level of autophagy and apoptosis [24]. Our previous investigation showed that miR-21-5p can activate the PI3K/Akt/mTOR signaling pathway, inhibit AECII autophagy and apoptosis, and improve HALI [25, 26]. However, it was not clarified whether the inhibitory effect of miR-21-5p on AECII apoptosis via PI3K/Akt/mTOR is related to the inhibition of autophagy. To further explore the relationship between mTOR pathway-mediated autophagy and AECII apoptosis in HALI, mTOR inhibitors, and activators were used in the current study to up- and downregulate autophagy and observe AECII apoptosis and lung injury in HALI models.

## Materials and Methods

### Materials and Reagents

Cell culture materials were obtained from NEST (Wuxi, China). 3% H_2_O_2_ was purchased from Guizhou Xinyuan Biotechnology Corporation (Guizhou, China). ROS assay kit was purchased from Thermo Fisher Scientific Corporation (Shanghai, China). Annexin V-FITC/ PI kit was purchased from BD Corporation. CCK-8 was obtained from Topscience (Shanghai, China). Rapamycin (Rapa), MHY1485, and Chloroquine (CQ) were purchased from MCE (Shanghai, China). TUNEL kit was purchased from Beyotime (Shanghai, China). Antibodies against LC3B, P62, mTOR, p-mTOR, caspase-3, and cleaved caspase-3 were obtained from CST (Shanghai, China), while β-actin antibody was from Proteintech (Wuhan, China).

### Cell Culture and Treatment

Primary AECII were extracted and cultured as previously described [27]. MLE-12 AECII were purchased from ATCC and cultured in DMEM/ F12 medium supplemented with 10% fetal bovine serum. The final concentration of 0.5-mM H_2_O_2_ is changed to serum-free medium when modeling is induced.

### Animals and Animal Model

Male SD rats (200–250 g, 8–10 weeks old) were purchased from Hunan Silaikejingda Experimental Animal Co., Ltd. Animal License No. SCXK (Xiang 2019-0004). The animal ethics has been approved by the Animal Experimentation Ethics Committee of Zunyi Medical University (Approval No. KLLY(A)-2020-040). Animal models we-re established by exposing SD rats to hyperoxia (90% oxygen concentration) for different times (0, 24, 48, and 72 h). Rapa and MHY1485 were administered via intraperitoneal injection prior to hyperoxia exposure.

### Transmission Electron Microscope (TEM)

Primary AECII with specific lamellar vesicles and Microvilli structures were observed by transmission electron microscopy.

### Immunofluorescence (IF) Staining

Primary AECII cells were cultured for 48 h in 6-well plates with cell crawlers, fixed with 4% paraformaldehyde for 15 min, closed with 5% BSA for 1 h, incubated overnight with primary antibody at 4 °C, washed 3 times with PBS, incubated for 2 h with secondary antibody, sealed with anti-fluorescence quencher containing DAPI, and then photographed.

### CCK-8

96-well plates were inoculated with MLE-12 cells (6000–8000/100 μl). CCK-8 reagent (100 μL per well) was added to incubate with the cells at 37 °C for 2 h and the absorbance at 450 nm was measured.

### Detection of ROS

MLE-12 was inoculated in a 6-cm dish and incubated for 48 h followed by the addition of 1 × ROS working solution to incubate for 1 h at 37 °C. ROS level was measured using flow cytometry.

### mRFP-GFP-LC3 Double-Tag Assay

MLE-12 was counted and inoculated in a 6-well plate with crawler for 24 h. mRFP-GFP-LC3 autophagic double-labeled adenovirus (titer of 1.26 × 10^10^ PFU/mL) was used to infect the cells for 8 h. 36 h later, the fluorescent intensity was observed. After designated treatments, the cells were fixed in 4% paraformaldehyde for 15 min and sealed for photographs.

### Detection of Apoptosis

Treated MLE-12 cells were inoculated in 6-well plates and incubated for 48 h and then digested with 0.25% EDTA-free trypsin for centrifugation and supernatant removal. After that, cells were resuspended with 1 × Binding Buffer (about 1 × 10^5^ cells per 100 μL) and 5-μL FITC-Annexin V and 5-μL PI dye were added to each 100-μL cell suspension to incubate for 15 min at room temperature in darkness. 200 μL of 1 × Binding Buffer per tube was added and apoptosis rate was analyzed by flow cytometry.

### TUNEL Staining

Lung tissue sections were dewaxed by oven at 60 ℃ for 15 min and then dewaxed again with xylene. Following hydration with ethanol and distilled water, the tissue sections were permeabilized with proteinase K at 37 ℃ for 30 min, washed 3 times with PBS, and then incubated with 100 μl of TUNEL staining solution at 37 ℃ for 1 h. The tissues were washed 3 times with PBS and sealed with DAPI anti-fluorescence quencher.

### HE Staining

Lung tissue sections were dewaxed, hydrated, and stained with hematoxylin for 15 min and eosin staining solution for 3 min. The pathological changes of the tissue sections were photographed and observed.

### Western Blotting

Western blot was performed following previous steps with indicated antibodies [25].

### Statistical Analysis

Statistical analysis was performed using SPSS 20.0 software and figures were generated with GraphPad prism 8.0.2. The normal distribution was determined by *K*–*S* test and normally distributed data were displayed as mean ± standard deviation (*x* ± *s*). *t* test and One-way ANOVA were used to compare two independent samples and multiple groups as appropriate. In case the variance was the same, the LSD method was used to compare two groups; if not, Dunnett’s T3 method was used. For the comparison between multiple experimental factors and groups, the analysis of variance of factorial design was performed. Differences were considered statistically significant at *P < 0.05.

## Results

### Hyperoxia Triggered Autophagy Activity In Vivo

**Fig. 1:**
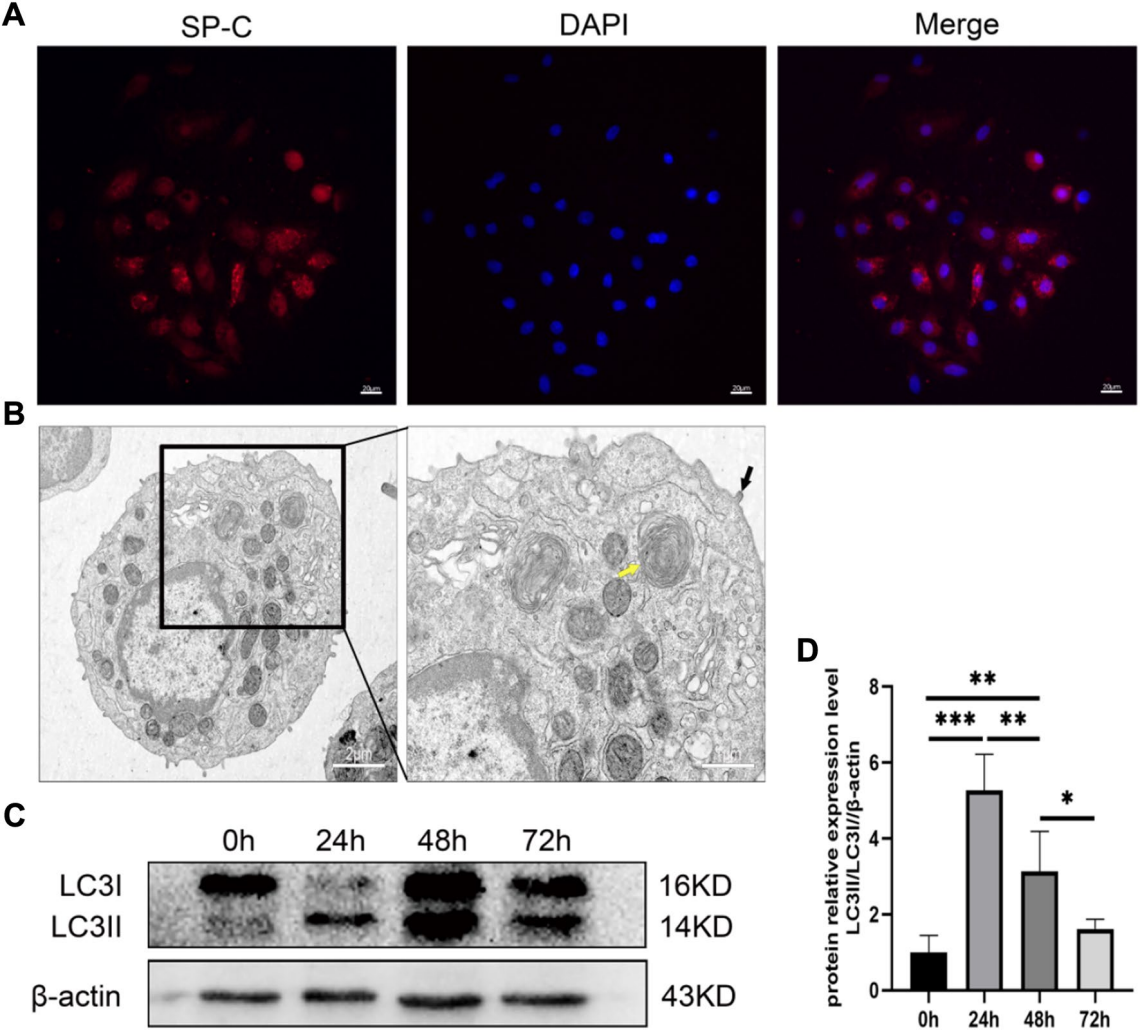
Hyperoxia can induce AECII autophagy in vivo. (A) AECII-specific protein SP-C staining. (B) TEM observation of AECII-specific structures. (C-D) Relative expression levels of LC3 at different times of hyperoxia exposure. *, *P* < 0.05; **, *P* < 0.01; ***, *P* < 0.001

To evaluate whether hyperoxia induces AECII autophagy, primary AECII was isolated and purified from hyperoxia-exposed SD rats. After isolation, the AECII was characterized as IF staining detected the positive staining of primary SP-C protein (Fig. 1A) and AECII-specific structures, including lamellar vesicles (yellow arrows) and microvilli (black arrows), were observed using TEM (Fig. 1B). Then, western blot detected the relative expression level of autophagy-related protein LC3 which showed relatively higher expression at 24 h and 48 h (Fig. 1C, D). These results suggested that hyperoxia stimulated autophagy in the AECII from hyperoxia-exposed SD rats.

### Hyperoxia Caused Lung Injury In Vivo

**Fig. 2:**
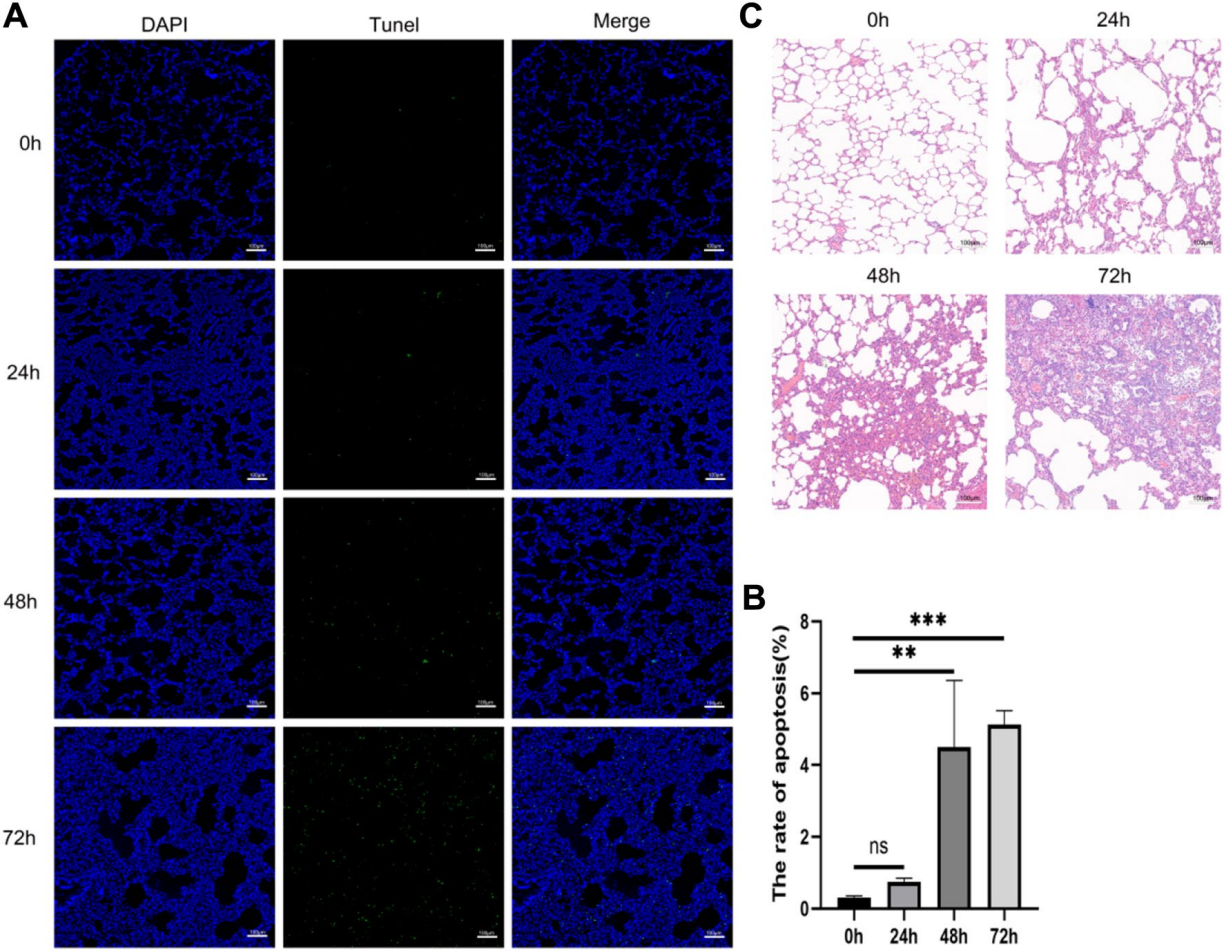
Hyperoxia can induce lung tissue apoptosis and lung injury. **A**, **B** Hyperoxia can induce apoptosis in lung tissue. **C** Hyperoxia can induce lung injury. **, *P* < 0.01; ***, *P* < 0.001

Next, the effect of hyperoxia on lung tissues was evaluated. TUNEL staining indicated that the apoptosis in lung tissues were remarkably enhanced at 48 h and 72 h (Fig. 2A, B). In addition, H&E staining results showed that hyperoxia caused various degrees of alveolar wall rupture, interstitial thickening, and inflammatory infiltration in lung tissues in a time-dependent manner (Fig. 2C). Taken together, hyperoxia exposure caused significant apoptosis and injuries in the lung tissues of model rats.

### H_2_O_2_ Induced Autophagy in MLE-12 Cells

**Fig. 3:**
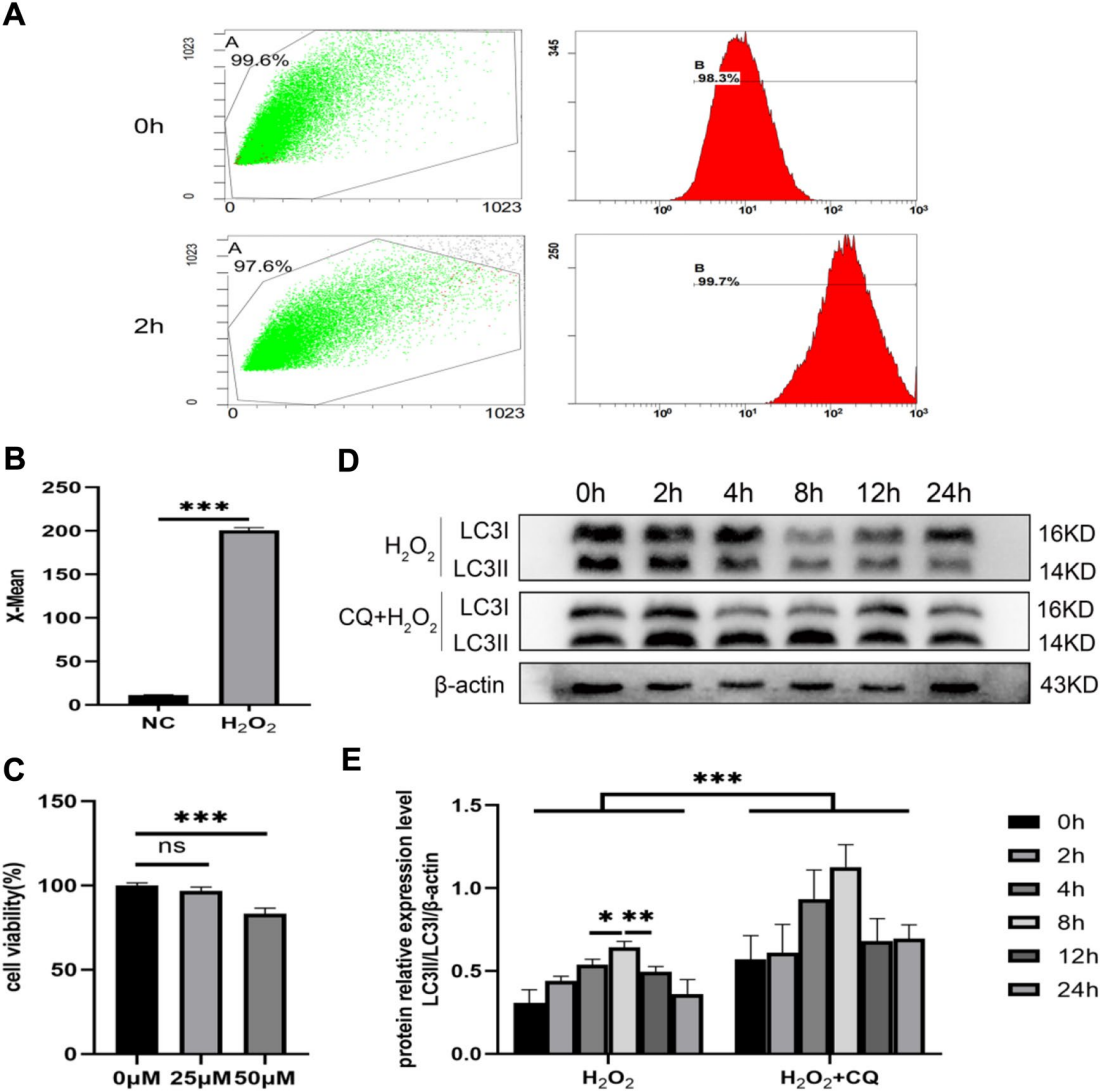
H_2_O_2_ induces MLE-12 autophagy in vitro. **A**, **B** Evaluate the effect of modeling with a final concentration of 0.5-mM H_2_O_2_. **C** CCK8 mapping CQ modeling concentration. **D**, **E** CQ-assisted determination of the relative expression levels of LC3 at different times of H_2_O_2_. ^ns^, *P* > 0.05; *,* P* < 0.05; ***, *P* < 0.001

To explore the relationship between autophagy and lung injury under hyperoxia, we conducted in vitro experiments in H_2_O_2_-stimulated MLE-12 cells. H_2_O_2_ at the concentration of 0.5 mM for 2 h was used to establish in vitro HALI model. The ROS level was significantly elevated following H_2_O_2_ treatment (Fig. 3A, B). To determine the participation of autophagy, the autophagy tool drug CQ was employed to treat H_2_O_2_-challenged MLE-12 cells. CQ at the concentration of 25 μM showed no cytotoxicity and was used for subsequent experiments (Fig. 3C). Western blot indicated that LC3 expression peaked at 8 h in both H_2_O_2_ group and CQ + H_2_O_2_ group, while the overall LC3 expression was higher in CQ + H_2_O_2_ group than that in the H_2_O_2_ group. (Fig. 3D, E). Briefly speaking, H_2_O_2_ activated autophagy activity in MLE-12 cells.

### H_2_O_2_ Inhibited mTOR to Trigger MLE-12 Autophagy and Apoptosis

**Fig. 4:**
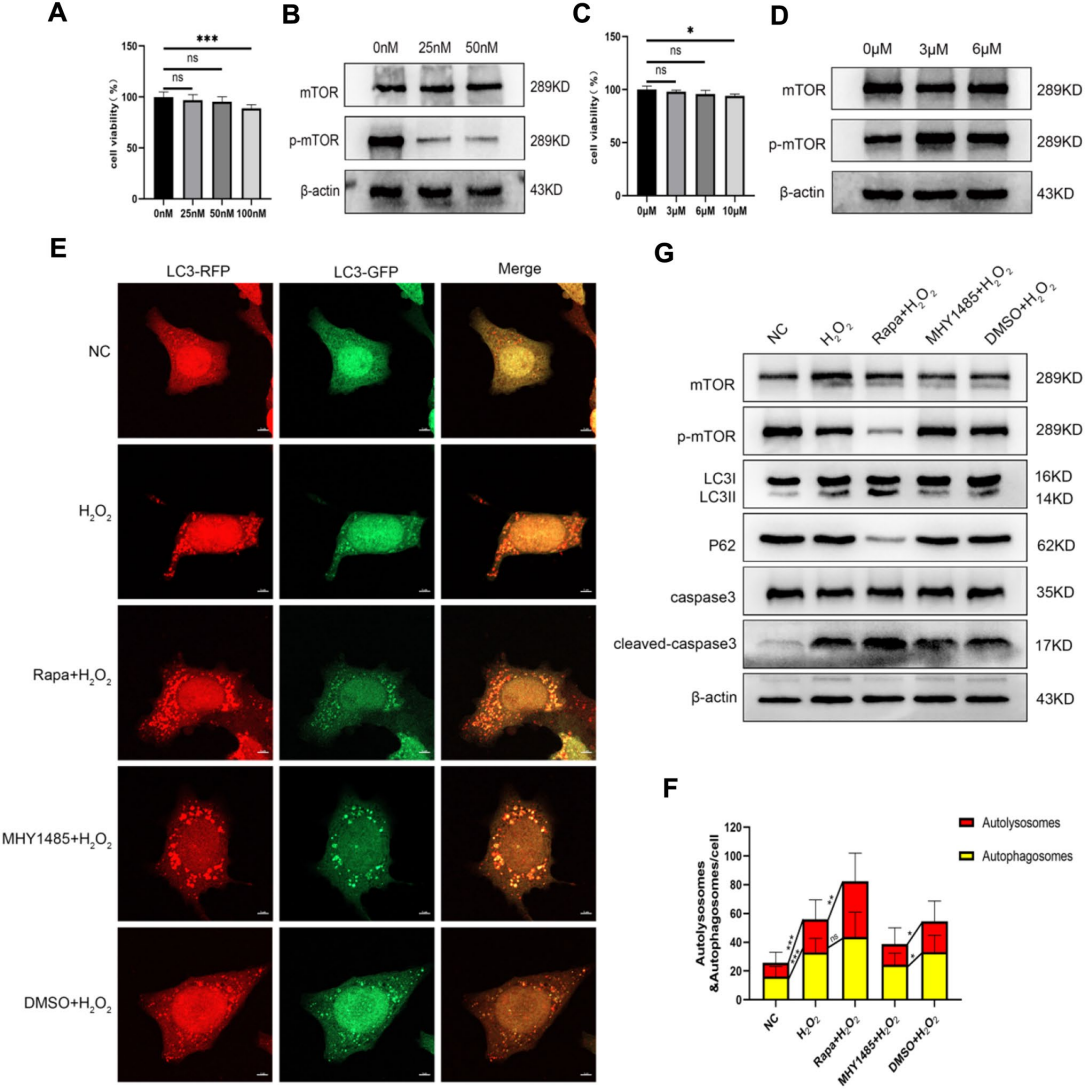
H_2_O_2_ can upregulate autophagy and apoptosis via mTOR. **A**–**D** CCK8, WB mapping mTOR inhibitor Rapa, and activator MHY1485 modeling conditions. **E**, **F** Autophagy double labeling to assess changes in autophagy levels. **G** WB assessment of relative expression levels of mTOR and p-mTOR; autophagy-related proteins LC3 and P62; and apoptosis-related protein caspase-3 and cleaved caspase-3. ^ns^, *P* > 0.05; *, *P* < 0.05; **, *P* < 0.01, ***, *P* < 0.001

**Fig. 5:**
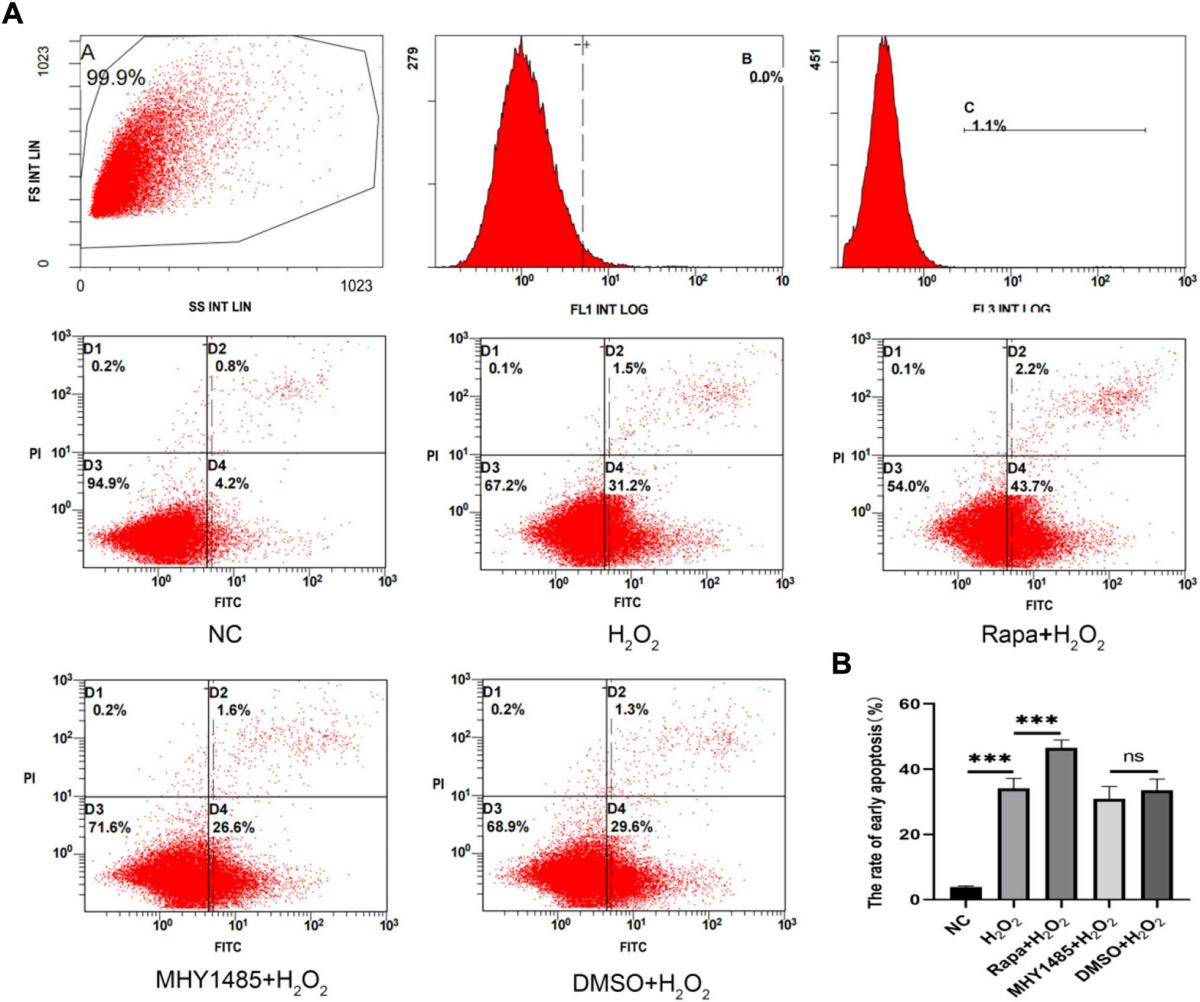
Flow cytometry explores early apoptosis. **A**, **B** Early apoptosis rate in each treatment group. ^ns^, *P* > 0.05; ***, *P* < 0.001

To explore the mechanism underlying the regulatory effect of H_2_O_2_ on autophagy and apoptosis in MLE-12 cells, the mTOR inhibitor Rapa (25 nM for 12 h) and activator MHY1485 (3 μM for 8 h) were used to mediate mTOR activity (Fig. 4A–D). Following the treatments, autophagy double labeling indicated that Rapa augmented the enhancement of autophagy activity stimulated by H_2_O_2_, whereas MHY1485 attenuated H_2_O_2_-triggered autophagy (Fig. 4E, F). Moreover, western blot suggested that H_2_O_2_ upregulated the expression of LC3 and cleaved caspase-3 but downregulated the level of p-mTOR and p62; meanwhile, Rapa functioned synergistically with H_2_O_2_, while MHY1485 abrogated the effects of H_2_O_2_ (Fig. 4G). The apoptosis detected by flow cytometry corroborated the expression change trend of cleaved caspase-3 among different treatment groups (Fig. 5A, B). In sum, H_2_O_2_ facilitated autophagy and apoptosis in MLE-12 cells by blocking mTOR activation.

### mTOR-Mediated Autophagy Regulated Hyperoxia-Induced AECII Apoptosis and Lung Injury In Vivo

**Fig. 6:**
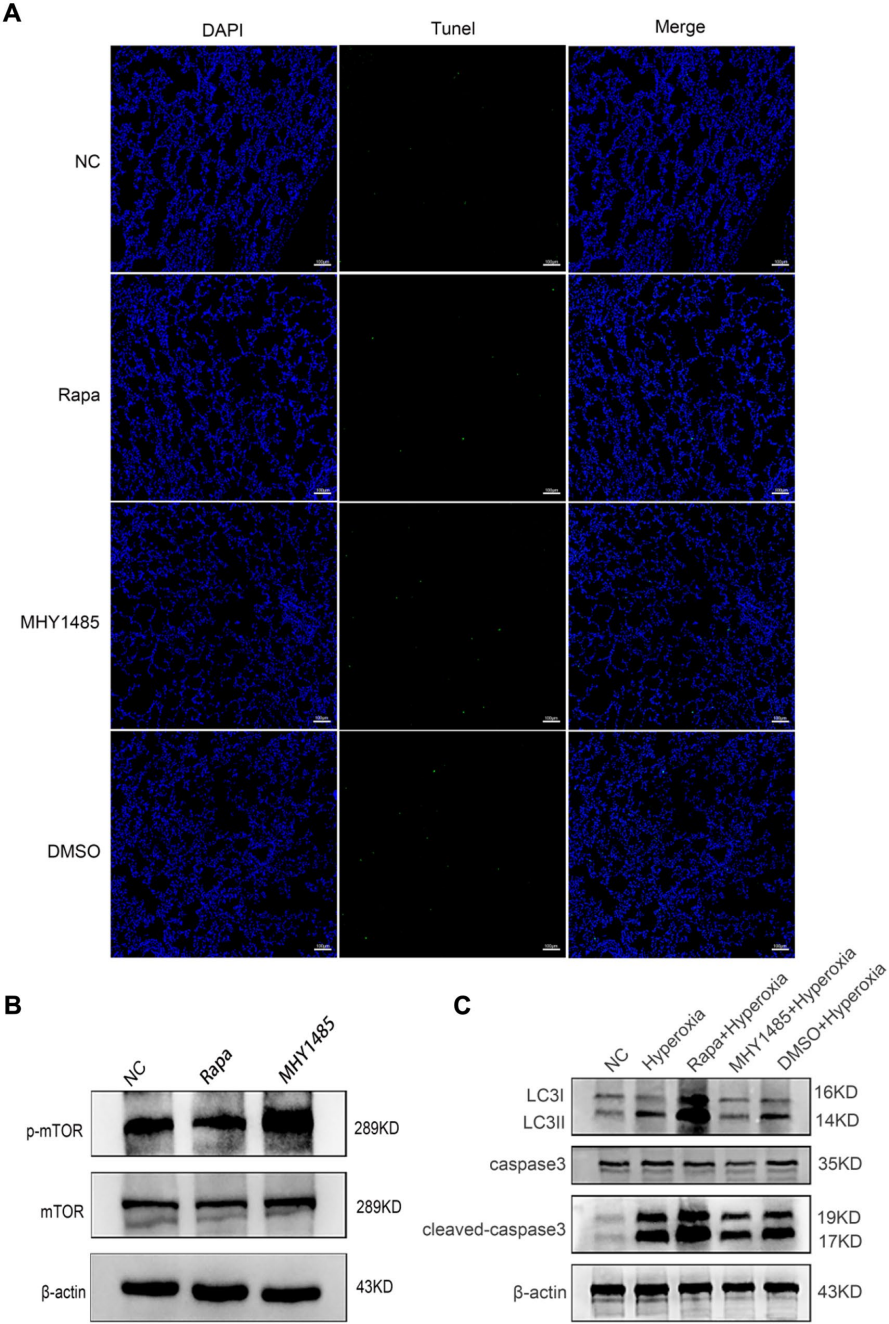

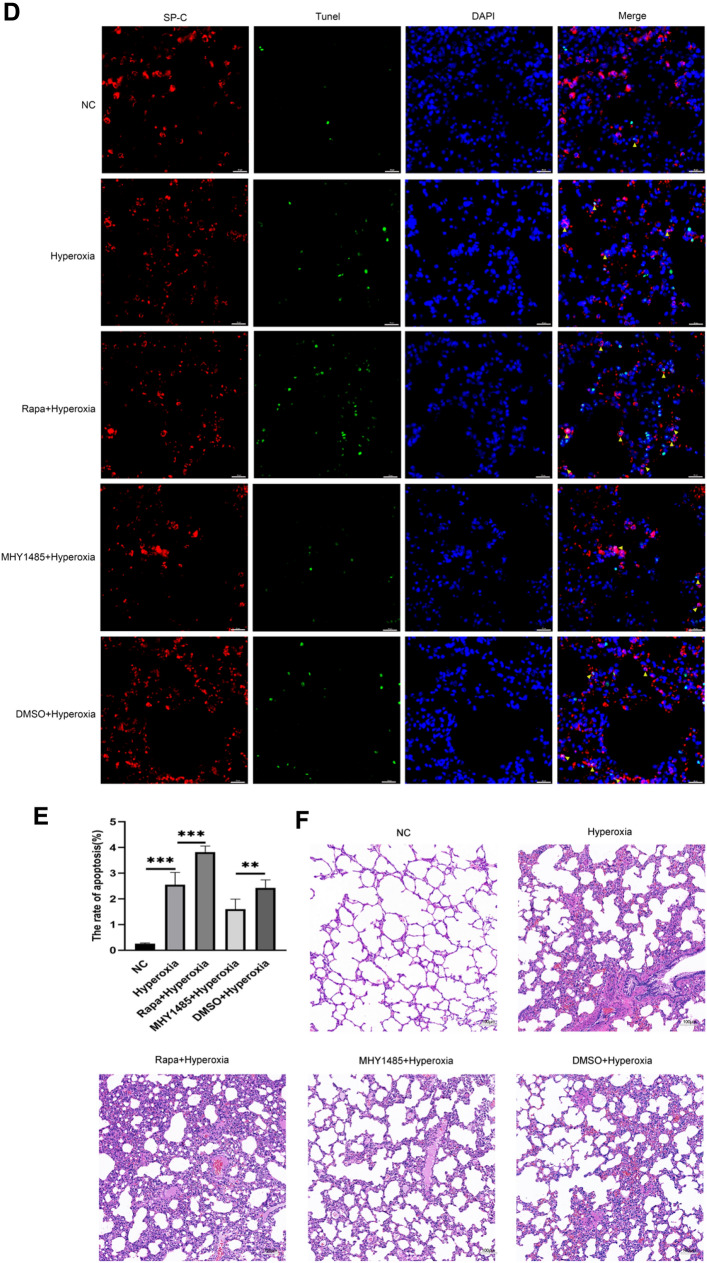
Assessing the regulation of apoptosis by autophagy via mTOR in vivo experiment. **A**, **B** Transperitoneal injection of rapamycin and MHY1485 can regulate mTOR in lung tissue and do not cause additional apoptosis in lung tissue. **C** Relative expression of autophagy- and apoptosis-related proteins in each group. **D**, **E** Double staining of TUNEL and SP-C to assess the apoptosis rate of AECII. **F** Degree of lung tissue damage in each group. **, *P* < 0.01, ***, *P* < 0.001

Finally, the effect of mTOR activity was investigated in hyperoxia-exposed rat model by administering Rapa or MHY1485 to SD rats through intraperitoneal injection as per described in previous studies [28–30]. TUNEL staining suggested that administration of Rapa or MHY1485 had no obvious effect on cell death in the lung tissues of treated rats when hyperoxia pretreatment was absent, but the phosphorylation of mTOR was effectively affected (Fig. 6A, B). Subsequently, the effects of Rapa and MHY1485 on the primary AECII isolated from hyperoxia-exposed rats were investigated. As demonstrated by western blot, hyperoxia-stimulated autophagy and apoptosis was further enhanced by Rapa while activating mTOR with MHY1485 ameliorated hyperoxia-induced autophagy and apoptosis (Fig. 6C). Similarly, TUNEL and HE staining of the lung tissues revealed that autophagy activation by Rapa aggravated hyperoxia-induced AECII apoptosis and lung injury, while inhibition of autophagy reduced AECII apoptosis and alleviated lung injury (Fig. 6D–F). To summarize, activated mTOR inhibited autophagy activity to ameliorate hyperoxia-induced lung injuries.

## Discussion

It has been widely recognized that providing additional oxygen therapy or exposing critically ill patients to hyperoxia carries potential hazards [31], one of which is hyperoxia-induced multi-organ damage [32, 33], especially lung injuries [34]. Increasing studies focused on the understanding of oxygen toxicity in lung tissues recent years. In 2019, Yao et al. reported that fatty acid oxidation ameliorated endothelial cell apoptosis and lung injury induced by hyperoxia in neonatal mice [35]. More recently, AKAP1 deficiency was reported to trigger mitochondrial dysfunction in HALI mouse model [36]. In another case, Kim et al. reported that NLRX1 depletion blocked pro-apoptotic pathway in HALI [37]. Despite the indisputable fact that hyperoxia can cause multi-organ damage, the exact mechanisms are complex and remain inconclusive.

It has been suggested that AECII autophagy and apoptosis may be one of the mechanisms in HALI [5, 19]. To explore the involvement of AECII autophagy in HALI, we first constructed in vivo HALI model by exposing SD rats to hyperoxia. HE staining showed that the lung tissues of model rats were injured and activated autophagy and enhanced apoptosis was observed in the primary AECII isolated from the model rats, suggesting the possible contributing effect of autophagy and apoptosis to HALI development. In vitro model was established by stimulating MLE-12 with H_2_O_2_ at a final concentration of 0.5 mM which was proved by our previous investigation to be effective in the induction of hyperoxic model in AECII [38]. The concentration of H_2_O_2_ used here is also consistent with previously published literature [39]. The in vitro results corroborated the data obtained from in vivo experiments that H_2_O_2_ stimulation induced autophagy and apoptosis in MLE-12 cells.

The mTOR pathway is a classical pathway that regulates autophagy and apoptosis under pathological conditions of organ damages. For example, ASPP2 upregulated mTORC1- and ERS-related proteins to inhibit autophagy and reduce apoptosis in TNF-α-induced hepatocyte injury [40]. Nano-copper induced testicular damage by stimulating autophagy and apoptosis through its suppression on AKT/mTOR signaling [41]. Intriguingly, mTOR pathway was proved to play controversial role in lung injuries. Yang et al. revealed that Isorhamnetin inhibited the mTOR pathway to alleviate LPS-induced acute lung injury [42]. Wang et al. discovered that Cinobufagin activated the p53/mTOR pathway to alleviate LPS-triggered acute lung injury by autophagy induction [43]. Moreover, autophagy activation can either increase or decrease apoptosis under pathological conditions [44, 45].

Based on these findings, we further investigated the participation of mTOR pathway in hyperoxia-induced autophagy and apoptosis in HALI models. The well-recognized mTOR inhibitor (Rapa) and activator (MHY1485) were introduced to treat model rats or MLE-12 cells. We found that hyperoxia or H_2_O_2_ can inhibit the phosphorylation of mTOR and upregulate AECII autophagy and apoptosis. With the addition of mTOR inhibitor, hyperoxia-induced AECII autophagy and apoptosis were further augmented while mTOR activator effectively attenuated the toxicity of hyperoxia by suppressing AECII autophagy and apoptosis.

In summary, hyperoxia-activated AECII autophagy level promoted apoptosis and lung injury by suppressing the mTOR pathway. This has contributed to the deepening of our understanding on the pathogenesis of HALI. However, the current study is limited in several aspects. First, the reduction of early apoptosis rate in the mTOR activation group in cell experiments was not obvious. Second, it was reported that autophagy had different effects on apoptosis at different time points [21]. The current study investigated the relationship between mTOR-mediated autophagy and apoptosis at one time point only.

## Data Availability

The datasets used and/or analyzed during the current study are available from the corresponding author on reasonable request.
